# Whole Genome Analysis of Ocular *Pseudomonas aeruginosa* Isolates Reveals Genetic Diversity

**DOI:** 10.1167/iovs.66.6.58

**Published:** 2025-06-18

**Authors:** Samantha Wellington Miranda, Camille André, Paulo J. M. Bispo, Michael S. Gilmore

**Affiliations:** 1 Department of Microbiology, University of Washington, Seattle, Washington, United States; 2 Department of Ophthalmology, Massachusetts Eye and Ear, Harvard Medical School, Boston, Massachusetts, United States; 3 Infectious Disease Institute, Boston, Massachusetts, United States; 4 Department of Microbiology and Immunobiology, Harvard Medical School, Boston, Massachusetts, United States

**Keywords:** pseudomonas aeruginosa, ocular infection, genomics, antibiotic resistance, virulence

## Abstract

**Purpose:**

*Pseudomonas aeruginosa* is a versatile pathogen and leading cause of ocular infections. Several large-scale analyses have identified epidemic clones of *P. aeruginosa* adapted to human infection, but few genomic studies have focused on ocular infection. We sought to determine whether specific clones of *P. aeruginosa* are associated with ocular infection and to characterize the pool of virulence factors and antibiotic resistance genes present in ocular isolates.

**Methods:**

Ninety-two non-duplicate *P. aeruginosa* isolates from patients with ocular infections were collected in our service. Whole genome sequencing was completed followed by analysis of phylogeny, key virulence factors, and phenotypic and genotypic antibiotic resistance.

**Results:**

We found genetic diversity among our isolates, with no predominant clonal group. Analysis of virulence factors showed that the majority of our isolates encode the exotoxin ExoS, in contrast to previous studies in which ocular isolates were predominantly ExoU-positive. We also found a high frequency of mutations in the quorum sensing receptors LasR and RhlR, adding to growing evidence that such mutations are common in multiple types of infection. Finally, we identified two isolates lacking genes for biofilm exopolysaccharide Psl biosynthesis, as well as within-host variation in alginate production. Consistent with the community origins of our isolates, we found a low rate of antibiotic resistance (<7%), with only two multidrug resistant isolates.

**Conclusions:**

Our study significantly increases the number of ocular *P. aeruginosa* genomes available for phylogenomic analyses and reveals that *P. aeruginosa* ocular infections at our institution are caused by a highly genetically diverse population.

P*seudomonas aeruginosa* is among the most frequently identified Gram-negative bacteria causing eye infections worldwide, especially in keratitis, and is the leading cause of contact lens–associated keratitis.^1^^–^^3^ Keratitis caused by *P. aeruginosa*, characterized by suppurative infiltration, tends to be more severe, as it progresses rapidly, which can lead to corneal perforation and melt, resulting in vision loss.^4^ *P. aeruginosa* has a relatively large genome that encodes many virulence factors, including toxins, adhesins, and proteases, that are important during ocular infection.^5^ For example, *P. aeruginosa* encodes a type III secretion system (T3SS) that delivers toxins to host cells, including exotoxin S (ExoS), leading to an invasive phenotype, and exotoxin U (ExoU), leading to a cytotoxic phenotype.^5^ Strains of *P. aeruginosa* typically encode ExoS or ExoU, but not both. The phospholipase activity of ExoU results in rapid host cell lysis and has been associated with more severe disease in animal models, as well as in human clinical trials.^6^^,^^7^ On the other hand, ExoS enables *P. aeruginosa*, which is typically an extracellular pathogen, to invade and replicate inside host epithelial cells,^8^ potentially providing a niche that is protected from the immune system and from antibiotics. Compared to patients infected with ExoU^+^ isolates, patients with ExoS^+^ infections have been found to take longer to recover and may uniquely benefit from the addition of corticosteroids to their treatment regimen.^7^ Thus, ExoU/ExoS status may result in different courses of infection and may also indicate different therapeutic approaches. Several proteases, including LasA,^9^ *P. aeruginosa* small protease (PASP),^10^ and protease IV,^11^ have also been shown to impact virulence in animal models of keratitis. Further, *P. aeruginosa* forms strong biofilms, in which an extracellular matrix composed of exopolysaccharides (Psl, Pel, and/or alginate), proteins, and DNA protects the bacteria from antibiotics and other stresses.^12^ Many of these virulence factors and processes are regulated by three interconnected quorum sensing systems, which link gene expression to cell density.^12^^–^^15^ *P. aeruginosa* encodes two acyl homoserine lactone quorum sensing systems, LasI–LasR and RhlI–RhlR, and a quinolone-based system called PQS. Although quorum sensing is important for pathogenicity in several animal models of lung and wound infection,^15^ the contribution of quorum sensing to *P. aeruginosa* virulence during ocular infection remains unclear. Some studies have found reduced bacterial burden and disease severity in animals infected with *P. aeruginosa* lacking the quorum sensing receptor LasR,^16^^,^^17^ but others have found no difference between ocular infection with wild-type and ∆*lasR* strains.^9^ This variability in outcome may be due to differences in infection model or in strain background, including variability in quorum sensing networks^18^ and/or quorum-regulated virulence factors.^12^^,^^13^

Genomics has given us high‐resolution tools to better understand the molecular basis for the propensity of *P. aeruginosa* to cause acute and systemic infections, as well as chronic wound and pulmonary infections. For example, in a recent large-scale study of *P. aeruginosa* isolates, Weimann et al.^19^ identified 21 epidemic clones that have caused a significant proportion of *P. aeruginosa* infections worldwide and determined genetic patterns of host-specific adaptation, particularly for clones associated with respiratory infections in people with the genetic disease cystic fibrosis. However, despite this and numerous other studies that have investigated the genomic epidemiology of *P. aeruginosa* in various infection types,^20^^–^^23^ studies using whole genome sequencing to investigate the epidemiology and molecular basis of ocular *P. aeruginosa* infections are more limited, with most investigations performed in small cohorts (<20 ocular isolates for whole genome sequencing studies).^24^^–^^26^ To fill these gaps, we performed in-depth genomic characterization of 92 non-duplicate *P. aeruginosa* isolates recovered from patients with ocular infections at a large academic hospital in the United States. Using whole genome sequencing we reconstructed the phylogeny of the isolates to provide a detailed picture of their population structure, determined the prevalence of clones across different diseases to evaluate potential niche adaptations and tropisms, and characterized their pools of antimicrobial resistance markers and virulence factors.

## Materials and Methods

This experimental laboratory investigation study was approved by the Mass General Brigham Institutional Review Board (MGB IRB; protocol 2019P001001). Protocols for collection of discarded isolates were approved by the MGB IRB (protocol 2021P000695) for prospective sampling, and the need for informed consent was waived. The study adhered to the tenets of the Declaration of Helsinki and was conducted in accordance with Health Insurance Portability and Accountability Act (HIPAA) regulations.

### Bacterial Isolates

*P. aeruginosa* ocular isolates were collected from 92 patients presenting with eye infections at the Massachusetts Eye and Ear (MEE) hospital in Boston, MA, from 2014 to 2017, for a total of 104 isolates. Redundant, duplicate isolates from the same patient were excluded from the final analysis, which included a single non-duplicate isolate for each patient (*N* = 92). Primary clinical specimens collected from multiple ocular infection sites were obtained by the attending ophthalmologist or resident following institutional guidelines and were submitted to the clinical microbiology laboratory for processing. Bacterial identification was performed using the MicroScan WalkAway system (Beckman Coulter, Brea, CA, USA) following the manufacturer's protocol. Isolates were stored at −80°C in Microbank cryopreservative tubes (Pro-Lab Diagnostics, Richmond Hill, ON, Canada). Frozen isolates were cultured on 5% sheep blood agar plates (BD Biosciences, San Jose, CA, USA) and incubated at 37°C.

### Genome Sequencing and Assembly

We performed whole genome sequencing on 104 isolates included in this study. Total DNA was purified using the DNeasy DNA extraction kit (QIAGEN, Hilden, Germany) from an overnight pure culture in 5 mL brain heart infusion (BHI) broth. DNA quality was verified on a Synergy 2 microplate reader (BioTek, Winooski, VT, USA) prior to quantification using an Invitrogen Qubit fluorometer and dsDNA High-Sensitivity assay kit (Thermo Fisher Scientific, Waltham, MA, USA). Library preparation for Illumina sequencing was carried out using the Nextera XT DNA Library Preparation Kit (Illumina, San Diego, CA, USA), according to the manufacturer's specifications. The quality and quantity of each sample library were measured on a TapeStation instrument (Agilent Technologies, Santa Clara, CA, USA). The genomes were sequenced as 2 × 150 bp or 2 × 250 bp reads on an Illumina MiSeq or HiSeq sequencer, according to the manufacturer's specifications, with a minimum depth of coverage of 30×. Sequence reads were assembled de novo using CLC Genomics Workbench (CLC Bio, Cambridge, MA, USA). This Whole Genome Shotgun project is publicly available through the National Center for Biotechnology Information (NCBI; BioProject no. PRJNA1209389).

### Phylogenetic Analysis

Reference genomes were obtained from the Bacterial and Viral Bioinformatics Resource Center (BV-BRC) database.^27^ A representative genome was included for each of the major sequence types (STs) identified as epidemic clones by Weimann et al.,^19^ selected first based on whether an isolate had been used in phenotypic studies and then by selecting the genome with the fewest contigs. We additionally included the commonly studied strains PAO1, DK2, and PAK, and we used the taxonomic outlier PA7 as an outgroup,^28^ resulting in 25 total reference genomes (Supplementary Table S2). To identify publicly available ocular *P. aeruginosa* genomes in the BV-BRC database, we used the following terms to query isolation source and host health: eye, cornea, keratitis, corneal, ocular, conjunctiva, lacrimal, conjunctivitis, contact lens, periocular, and endophthalmitis.

Genome sequences for the MEE isolates were uploaded to BV-BRC and annotated using RAStk^29^ in the BV-BRC workspace to assign genes to PathoSystems Resource Integration Center (PATRIC) genus-specific families (PGFams).^30^ A maximum-likelihood phylogenetic tree was constructed for our isolates and reference genomes using the BV-BRC Bacterial Phylogenetic Tree Service on default settings in which gene and protein sequences for 100 single-copy PGFams are aligned using BioPython^31^ or MAFFT^32^ and a phylogenetic tree is generated using RAxML^33^ with 100 rounds of rapid bootstrapping.^34^ In the case of multiple isolates from a single patient, the genome with the fewest contigs was included in the phylogenetic tree. The phylogenetic tree was visualized using the interactive Tree of Life (iTOL).^35^ To analyze average nucleotide identity (ANI), genome sequences were imported to the KBase workspace^36^ and ANI was computed for every pairwise set of genomes using FastANI.^37^

### Virulence Factor Analysis

The BV-BRC genome annotation pipeline includes virulence factor analysis using the virulence factor database (VFDB),^38^ providing an inventory of all putative virulence genes for each isolate. Each genome was additionally queried for two exotoxins, ExoU and ExoS, and for four quorum sensing proteins, LasR, RhlR, LasI, and RhlI, using BLASTp^39^ in BV-BRC and sequences from PAO1 as references, except for ExoU, for which the PA14 sequence was used as a reference because PAO1 does not encode ExoU. Variant analysis was performed in the BV-BRC workspace using MUSCLE^40^ to align the sequences for LasR, RhlR, and LasI to the PAO1 sequences and the sequences for RhlI to the PAK sequence. The sequences for variants with apparent deletions or truncations were manually inspected to confirm the presence of a nonsynonymous mutation. Truncated sequences present at the end of contigs were excluded from analysis. To identify the genes related to the major biofilm exopolysaccharides Psl, Pel, and alginate, we used the subsystems tool^41^ in the Comparative Systems service in BV-BRC to detect the presence or absence of these biosynthetic pathways in our isolates. For isolates with no *psl* genes detected by the Comparative Systems service, we used BLASTp with PAO1 protein sequences as a reference to query each gene in the pathway and confirm their absence. To analyze MucA sequences, we used BLASTp with the PAO1 sequence as a reference. To determine mucoid status, select isolates were streaked on 5% sheep blood agar plates and visually assessed for colony morphology.

### Antibiotic Susceptibility Testing

Routine antimicrobial susceptibility testing was performed using the MicroScan WalkAway system following the manufacturer's protocol. Quality assurance was performed by concurrently testing Clinical and Laboratory Standards Institute (CLSI)-recommended strains. Susceptibility breakpoints are not available for topical therapy of ocular surface infections using eye drops or for antibiotics given as intraocular injections to treat endophthalmitis. Because of that, antimicrobial susceptibility data from ocular bacteria are routinely interpreted using CLSI-approved interpretive breakpoint criteria that are developed for non-ocular infections treated with oral or intravenous antibiotics,^42^ and these breakpoints were applied in our study to classify bacterial isolates into susceptible and non-susceptible. The panel of antibiotics and concentrations tested in the MicroScan WalkAway system are summarized in Supplementary Table S3. The following antibiotics from three different classes were tested: ciprofloxacin and levofloxacin (fluoroquinolones); aztreonam, cefepime, ceftazidime, imipenem, and piperacillin/tazobactam (beta-lactams); and amikacin, gentamicin, and tobramycin (aminoglycosides). Non-susceptibility rates were calculated by combining the rates of intermediate and resistant isolates. Multidrug resistance (MDR) was defined as resistance to three or more classes of antibiotics.

### Prediction of Sequence Types and Antibiotic Resistance Genes

We used the Center for Genomic Epidemiology pipeline to obtain confirmation of species identification. We used the multilocus sequence typing (MLST) Center for Genomic Epidemiology pipeline to obtain STs based on the following seven housekeeping genes (from the pubMLST database)^43^: *acsA*, *aroE*, *guaA*, *mutL*, *nuoD*, *ppsA*, and *trpE*. ResFinder^44^ and Comprehensive Antibiotic Resistance Database (CARD)^45^ algorithms were used to identify the pool of acquired antibiotic resistance genes in each genome.

## Results

### Bacterial Population

A total of 92 consecutive and non-duplicate *P. aeruginosa* isolates causing eye infections, including keratitis (*n* = 74), lacrimal system infections (*n* = 7), periocular infections (*n* = 6), conjunctivitis (*n* = 3), and endophthalmitis (*n* = 2) (Table 1), were recovered from patients treated at MEE from 2014 to 2017. Most of these patients were female (62%) (Table 1). Age at presentation ranged from 14 to 92 years (median, 57 years). In our population, patients presenting with *P. aeruginosa* ocular infections often had a history of contact lens wear (29.3%) (Table 1), all of whom had keratitis (Supplementary Table S1). Contact lens wear, however, was not reported in the medical records for the majority of patients and is therefore unknown for those patients. In addition, we found that eight patients were co-infected with another bacterial species in their eyes, including *Serratia marcescens* (*n* = 3), *Staphylococcus aureus* (*n* = 4) and *Enterococcus faecalis* (*n* = 1) (Supplementary Table S1). Among patients with polymicrobial infections, only three of eight had keratitis (37.5%), compared to 74 out of 92 patients (80.4%) who presented with keratitis in the entire cohort. The frequency of polymicrobial infection among keratitis patients (4.1%) was significantly lower than the frequency among non-keratitis patients (26.3%) (*P* = 0.002, two-tailed *Z*-test).

**Table 1.** Isolate Sources

	*n* (%)
Sex	
Female	57 (62.0)
Male	35 (38.0)
Contact lens use (yes)	27 (29.3)
Diagnosis	
Keratitis	74 (80.4)
Lacrimal system infection	7 (7.6)
Periocular infection	6 (6.5)
Conjunctivitis	3 (3.3)
Endophthalmitis	2 (2.2)

### Phylogenetic Analysis of Ocular Isolates

**Figure 1.**
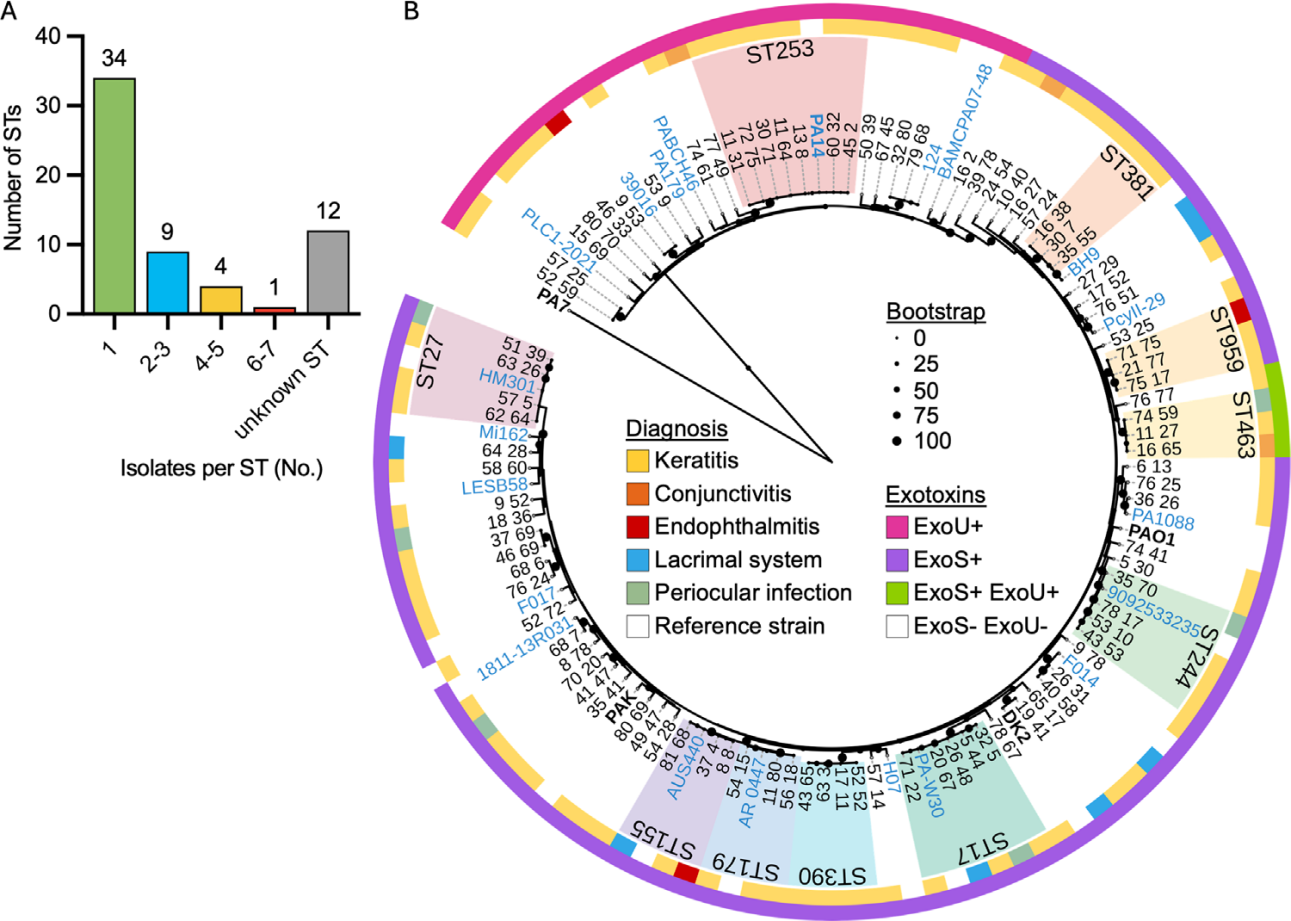
(**A**) Counts of STs containing the indicated number of isolates. (**B**) Maximum-likelihood phylogenetic tree of ocular isolates from MEE and reference genomes. Common reference genomes are indicated by *bold labels*. Reference genomes for epidemic clone groups are indicated by *blue labels*. STs with three or more isolates are indicated by *colored regions*. Patient diagnosis for each isolate is indicated by *colors on the inner ring*, and the presence of the genes encoding ExoS and/or ExoU is indicated by *colors on the outer ring*. *Black circles* on tree branches correspond to bootstrap values.

To determine whether specific *P. aeruginosa* clones are associated with ocular infection, we performed genomic analyses of our isolate collection. The isolates belonged to 48 multilocus STs,^46^ with ST being unknown for 12 isolates. Most isolates (*n* = 34) belonged to a unique ST, indicating a high level of genetic diversity among ocular isolates in this collection (Fig. 1A). Thirty-six of the isolates (39%) shared an ST with one of the recently identified “epidemic clone” groups.^18^ Among the 12 isolates with “unknown” STs, three of them (11_27, 64_28, and 9_78) were classified as “unknown” because of incomplete loci, eight of them (17_52, 27_29, 39_78, 50_39, 53_25, 57_14, 65_17, and 76_24) because of a locus combination with 6/7 loci match, and one of them (15_69) because of a novel allele in *mutL*. We also assessed ANI for every pairwise combination of isolates. Using a criterion of 99.988% ANI,^24^^,^^47^ no isolates from our collection were identified as clonal, except for isolates collected from the same patient. For each patient from which we collected multiple isolates, those isolates formed clonal groups (Supplementary Fig. S1), with the exception of isolates 43_65 and 40_24, which had an ANI of 99.9767%, and isolates 26_31 and 26_32, both of which had a high number of contigs and were therefore not analyzed for clonality. Overall, the majority of the MEE isolates belonged to distinct genetic lineages, with no predominant clonal lineages evident in the collection. A maximum-likelihood phylogenetic tree for our isolates along with 25 reference genomes (Supplementary Table S2) was constructed and shows that the isolates could be divided into two major groups, with group 1 containing reference strains PAO1 and PAK and group 2 containing PA14, consistent with other genomic analyses of strain diversity in *P. aeruginosa*.^48^^–^^50^ There was no clear phylogenetic clustering of isolates based on infection type (Fig. 1B).

### Virulence Factor Alleles Among Ocular Infection Isolates

*P. aeruginosa* encodes numerous toxins and virulence factors. On average, 255 virulence-associated genes were detected in each non-duplicate isolate using the Virulence Factor Database (VFDB)^38^ as a reference (range, 230–297 genes per isolate), excluding isolate 13_8, which had too many contigs for robust analysis. Here, we focus on three virulence-associated systems. The first is the T3SS through which multiple exotoxins are delivered to host cells. The majority of *P. aeruginosa* isolates encode the T3SS toxin ExoS or ExoU, but not both, leading to invasive or cytolytic phenotypes, respectively.^49^ Previous studies of ocular isolates have found a relatively high frequency of ExoU^+^ cytolytic isolates.^51^^–^^53^ Among the isolates sequenced in this study, 66 encoded ExoS (71.7%), 21 encoded ExoU (22.8%), four encoded both ExoU and ExoS (4.3%), and one isolate had neither toxin in its genome sequence (1.1%) (Table 2). All *exoU*^+^ *exoS*^–^ isolates clustered with group 2 strains, whereas the *exoS*^+^ isolates clustered with group 1 strains (Fig. 1B). The four isolates encoding both ExoU and ExoS came from diverse infection sources (keratitis, periocular disease, and conjunctivitis) and clustered in a single clade, with three of the four isolates belonging to ST463 (Fig. 1B). Of note, all eight of the isolates from patients with co-infection were *exoS*^+^.

**Table 2.** Virulence Factors

	*n* (%)
Exotoxins	
*exoS^+^*/*exoU^−^*	66 (71.7)
*exoS^−^*/*exoU^+^*	21 (22.8)
*exoS^+^*/*exoU^+^*	4 (4.3)
*exoS^−^*/*exoU^−^*	1 (1.1)
Quorum sensing variant	
LasR	15 (17.8)
LasI	1 (1.1)
RhlR	11 (12.4)
RhlI	4 (4.5)
Biofilm	
*pel^+^*	92 (100)
*psl*^+^	89 (97.8)
MucA variant	10 (9.6)

Clinical isolates of *P. aeruginosa* also have variability in their quorum sensing genes. Numerous studies have found that the quorum sensing receptor gene *lasR* is frequently mutated in *P. aeruginosa* isolates from chronic infections.^18^^,^^54^^,^^55^ Recent studies indicate that *lasR* mutations may also be common in *P. aeruginosa* isolates from ocular infections.^56^^,^^57^ To better understand the prevalence of quorum sensing mutations during eye infection, we analyzed the quorum sensing genes of isolates in our collection. We found that *lasR* mutations were common among our isolates and were variable, with no mutations shared by isolates from different patients. Of the 92 patients, nine harbored isolates that encoded an amino acid substitution in LasR and six had isolates that encoded truncation, deletion, or frameshift mutations relative to the laboratory strain PAO1 (Table 2, Supplementary Table S4). Mutations in *rhlR* were also common among our isolates. Seven isolates encoded an amino acid substitution in RhlR, and four had premature stop codons, deletions, or frameshift mutations relative to the PAO1 sequence. Two isolates, 32_80 and 79_68, had the same S97R substitution in RhlR. These isolates belonged to ST309 and were in a clade with reference strain 124, which also has the S97R substitution. Eight isolates were not analyzed for LasR, and three isolates were not analyzed for RhlR because their sequences were at the end of a contig and incomplete. Overall, 17.9% of isolates analyzed (15/84) encoded a mutation in LasR, and 12.4% of the isolates analyzed (11/89) encoded a mutation in RhlR (Table 2).

Nonsynonymous changes in the quorum sensing signal synthase genes *lasI* and *rhlI* were relatively uncommon (Table 2, Supplementary Table S4). A single isolate, 57_14, had a *lasI* mutation, which resulted in a 17-amino-acid deletion. *P. aeruginosa* strains have high diversity in the RhlI sequence at amino acids 62, 83, and 127. Because PAO1 is a relative outlier for the RhlI sequence, we used PAK as the reference strain for RhlI analysis.^55^ Thirty-one isolates had an amino acid substitution in RhlI relative to PAK, and four isolates could not be analyzed because their *rhlI* sequences were at the end of a contig or had uncertain sequences. The seven isolates belonging to ST253 had a Rh1I sequence identical to that of PA14, which is also ST253 (Supplementary Table S4). Excluding these isolates, as well as isolates that encoded only E83D and/or G62S substitutions, which are also found in PAO1, four of 88 isolates analyzed encoded a RhlI variant (4.5%). Three of these isolates belonged to ST463 and encoded the substitution P159S, and all four of these isolates also possessed a mutation in *lasR* or *rhlR* (Supplementary Table S4). In sum, when common RhlI alleles were excluded from the analysis, 27 of 92 isolates (29%) had a nonsynonymous mutation in the quorum sensing genes *lasR*, *lasI*, *rhlR*, and/or *rhlI*.

Finally, we assessed genes required for the synthesis of the major biofilm exopolysaccharides Psl, Pel, and alginate. Of two common laboratory strains, PAO1 encodes the genes necessary to produce all three exopolysaccharides, whereas PA14 has a three-gene deletion in the *psl* operon and uses Pel as the primary structural scaffold for biofilm formation.^58^ All isolates in our collection encoded the genes required for Pel and alginate production; three isolates were missing all or some genes for Psl synthesis. For isolates 11_31 and 16_65, we were unable to detect any of the *psl* genes. Isolate 80_69 encoded the full pathway except *pslA* and *pslB*, but the gene cluster is at the end of a contig; thus, it is possible that this isolate encoded these genes but they were missing from the draft genome. Overall, 89 of 91 isolates (97.8%) encoded the genes required for Psl synthesis (Table 2), with isolate 80_69 excluded from analysis due to ambiguity.

Alginate production is regulated by σ^22^ and its cognate anti-sigma factor MucA. Many clinical isolates, particularly from chronic lung infections of people with cystic fibrosis, harbor loss-of-function mutations in *mucA*, resulting in overproduction of alginate and a mucoid phenotype.^59^ Of all 104 isolates in our collection, 10 encoded a nonsynonymous mutation in *mucA* (9.6%) (Table 2, Supplementary Table S5). These 10 isolates were collected from eight unique patients. Two isolates encoded the common *mucA22* mutation, a deletion at *mucA* base 430 resulting in a frameshift and truncation of MucA. We also found intra-host variability in MucA sequences. Among a group of six isolates collected from a patient with periocular infection, two isolates (16_30 and 8_78) had identical mutations in *mucA*, but four isolates (12_48, 14_41, 16_29, and 3_62) were wild-type at this locus. Similarly, a patient with a lacrimal system infection had one isolate with a *mucA* mutation (17_52) and one with wild-type *mucA*. To confirm the functional relevance of these mutations, we assessed the mucoid status of groups of isolates from patients who harbored mixtures of wild-type and *mucA* mutants. Consistent with genomic data, isolates that harbored mutations in *mucA* were mucoid but those with wild-type *mucA* were non-mucoid (Supplementary Table S5). For all other patients with multiple isolates, the isolates were either uniformly *mucA* mutants or wild-type.

### Phenotypic and Genotypic Antimicrobial Resistance

**Figure 2.**
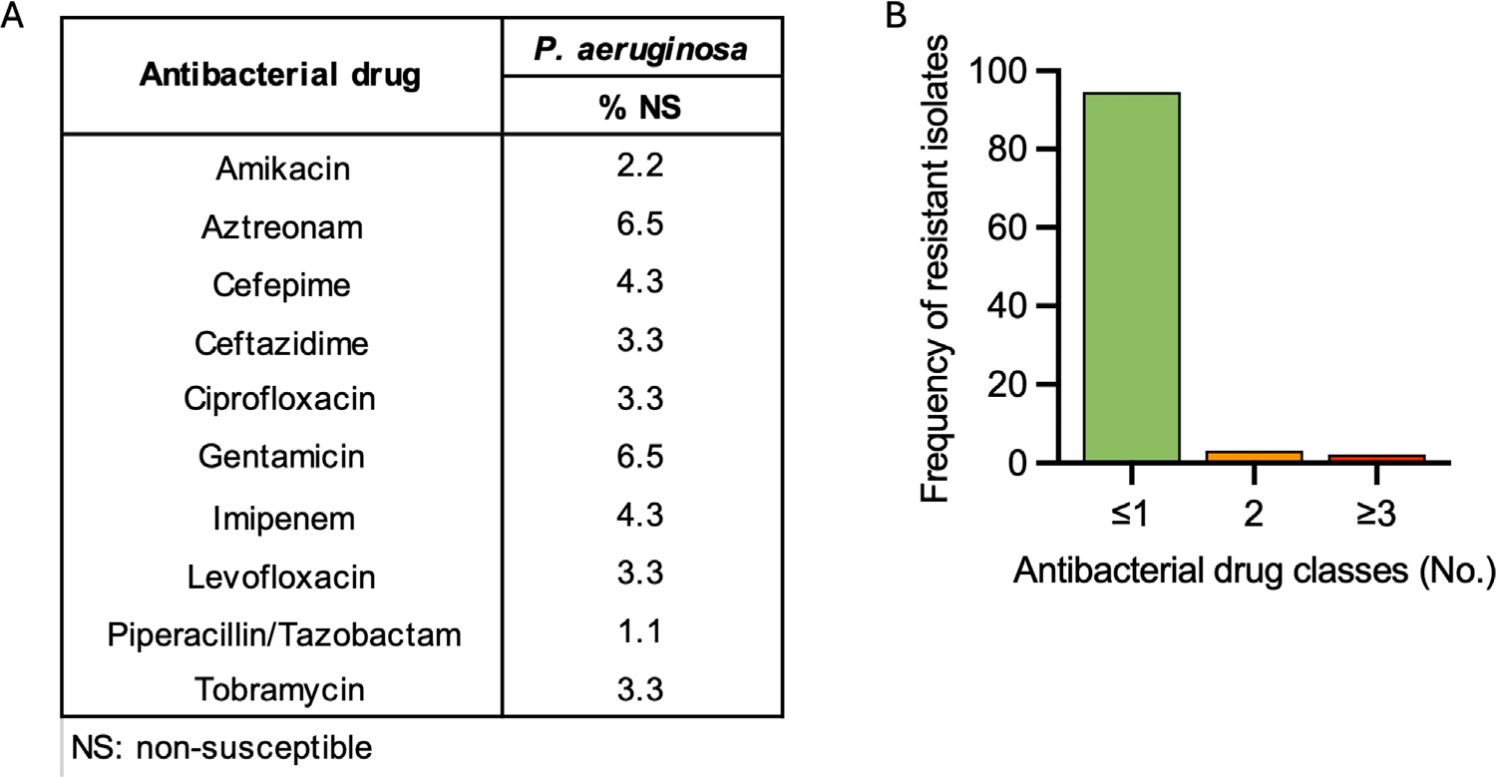
(**A**) Antibiotic resistance profiles for ocular *P. aeruginosa* isolates. (**B**) Concurrent resistance to antibiotics among ocular *P. aeruginosa* isolates.

To determine the rates of antibiotic resistance and the drugs that would optimize treatment outcomes of ocular *P. aeruginosa* infections based on in vitro susceptibility profiles, we tested the susceptibility of all isolates against a panel of commonly used antibiotics. We found low rates of resistance (<7%) among *P. aeruginosa* isolates to clinically important antibiotics that are often used for treatment of ocular infections, such as fluoroquinolones (3.3%), tobramycin (3.3%), and ceftazidime (3.3%) (Fig. 2A, Supplementary Table S1). Only two isolates (2.2%) were MDR (resistant to three or more classes of antibiotics) (Fig. 2B), belonging to ST235 (*n* = 1) and ST395 (*n* = 1).

To characterize the genotypic antibiotic resistance profile of *P. aeruginosa* causing eye infections, we used their draft genomes to identify the pool of acquired resistance genes carried by these isolates using the CARD^45^ and ResFinder^44^ databases. The most common acquired antibiotic resistance genes in our ocular *P. aeruginosa* population were the aminoglycoside resistance gene *aph(3′)-llb* (98.9%), beta-lactam resistance genes *blaOXA-50* (100%) and *blaPAO* (98.9%), chloramphenicol resistance gene *catb7* (100%), and fosfomycin resistance gene *fosA* (98.9%) (Table 3, Supplementary Table S1).

**Table 3.** Acquired Antibiotic Resistance Genes in *P. aeruginosa* Isolates Causing Eye Infections

	Antibiotic Resistance Genes, *n* (%)													
	AS					BL		CLP		FOS	TET		DP	
Species	*aph(3′)-llb*	*ant(2″)-Ia*	*aph(3″)-Ib*	*aph(6)-Id*	*ant(3″)*	*blaOXA-50*	*blaPAO*	*catb7*	*cmlA1*	*fos(A)*	*tet(C)*	*tet(M)*	*sul1*	*dfrA*
*P. aeruginosa* (*N* = 92)	91 (98.9)	1 (1.1)	1 (1.1)	2 (2.1)	1 (1.1)	92 (100)	91 (98.9)	92 (100)	1 (1.1)	91 (98.9)	1 (1.1)	1 (1.1)	3 (3.3)	2 (2.2)

AS, aminoglycosides; BL, beta-lactams; CLP, chloramphenicol; DP, diaminopyrimidine; FOS, fosfomycin; TET, tetracycline.

## Discussion

Although numerous genomic analyses of *P. aeruginosa* have been conducted, relatively few have focused on ocular infection. For example, when we queried the BV-BRC database,^27^ which contains >15,000 *P. aeruginosa* genomes, we found only 113 genomes with metadata consistent with being an ocular *P. aeruginosa* isolate, which is dwarfed by the several thousand publicly available genome sequences for isolates from lung infections of people with cystic fibrosis.^19^ Our analysis of 104 isolates from 92 unique patients is one of the largest whole genome studies of ocular *P. aeruginosa* isolates to date, enabling robust analyses of phylogeny, virulence factors, and antibiotic resistance genes.

We first sought to understand whether certain genotypic groups of *P. aeruginosa* are associated with ocular infection. Bacteria are often grouped into STs based on their alleles at six or more genes and are further grouped into “clonal complexes” that consist of closely related STs likely to have descended from a common ancestor.^43^ Several *P. aeruginosa* STs have been found to be associated with human infection and in some cases are also associated with increased virulence and/or antibiotic resistance.^21^ A recent analysis identified 21 *P. aeruginosa* clonal complexes that are highly prevalent, globally distributed, and more likely to be found in human infection than in the environment, leading them to be called “epidemic clones.”^19^ Although clonal complexes contain related STs, the term “strain” is applied to more closely related isolates that have identical or nearly identical genome sequences. As with clonal complexes, the definition of strain can vary, but it was recently proposed that whole genome ANI values of 99.99% be used to define strains.^47^ *P. aeruginosa* outbreaks can be caused by single strains of bacteria, often linked to a contaminated environmental source.^60^^,^^61^ The terms “strain” and “clone” are often used interchangeably, making it difficult to distinguish between studies of relatively broad clonal complexes versus more highly related strains.

Previous studies have suggested there are “epidemic clones” among *P. aeruginosa* ocular isolates,^51^^,^^53^^,^^62^ whereas others have found little evidence for dominant clones in ocular infection.^52^ Some of the discrepancy in these findings is likely related to each study's chosen definition of “clone.” The studies identifying clonal clusters among keratitis isolates employ a broad definition of the term, encompassing isolates with similar but non-identical STs. In these studies, the isolates have had a variety of STs with no single ST dominating. A small number of studies have found evidence for more closely related isolates causing keratitis in multiple patients. For example, 21 ocular isolates from two hospitals in India were found to belong to one of two STs and to share identical LasR substitutions within each of the STs, indicating a high likelihood of endemic epidemic strains.^56^ Likewise, whole genome sequencing of 13 ocular isolates showed that five isolates from a single hospital in India shared ≥99.98% ANI, again suggesting the presence of an epidemic strain.^24^ Although 39% of our isolates belonged to an ST consistent with one of the 21 recently identified epidemic clonal complexes,^19^ there was not a dominant ST within our collection. Further, using pairwise ANI for all genomes in our study, we found no evidence for an epidemic strain.

Population genomics studies have consistently shown that *P. aeruginosa* clusters into two major phylogroups: group 1 containing the reference strains PAO1, PAK, and DK2, and a relatively smaller group 2 containing PA14.^48^^–^^50^ It has been found that group 1 strains primarily encode the exotoxin ExoS, whereas group 2 strains encode ExoU.^49^ Among our isolates, 76% were ExoS^+^ and clustered with group 1 reference strains. This contrasts with previous studies in which keratitis isolates from the United Kingdom and India had a relatively high frequency of ExoU positivity, ranging from 47% to 62%.^51^^–^^53^^,^^62^ In these studies, the keratitis isolates primarily clustered with the reference strain PA14.^53^^,^^62^ The lower rate of ExoU^+^ isolates in our study is consistent with the majority of our isolates belonging to phylogroup 1. Other studies of ocular isolates from the United States and India have found similarly high frequencies of ExoS^+^ isolates, ranging from 73% to 84%,^7^^,^^63^^,^^64^ and a study from Japan reported a frequency of 56.5% *exoS^+^* ocular isolates.^65^ Thus, there may be regional or temporal diversity in the relative frequency of ExoU^+^ and ExoS^+^ *P. aeruginosa* isolates causing ocular infection.

*P. aeruginosa* clinical isolates also display variability in their quorum sensing systems LasI–LasR and RhlI–RhlR, which regulate several *P. aeruginosa* virulence factors.^5^^,^^12^^–^^15^ Despite the importance of quorum sensing for virulence in multiple infection models, *P. aeruginosa* isolates from chronic lung and wound infections frequently contain loss of function mutations in *lasR*.^18^^,^^54^^,^^55^ Recent evidence indicates that *lasR* mutations are also common in acute infections and potentially among environmental strains,^55^^,^^66^ and high frequencies of LasR loss of function (22%–26%) have been found in ocular isolate collections from the United States and India.^56^^,^^57^ Among our isolates, 17.8% harbor a nonsynonymous mutation in *lasR*, with a relatively high prevalence of frame shift, truncation, and deletion mutations likely to result in loss of function. Further, two of the missense mutations in our isolates have previously been found to result in LasR loss of function.^56^^,^^67^ The LasR alleles in our isolates were variable, with no isolates sharing a mutation except when collected from the same patient. This is consistent with a previous study from the United States^57^ and is also typically the case for LasR mutants isolated from people with cystic fibrosis.^18^^,^^55^^,^^67^ This suggests that, in our population, *lasR* mutations were present in a variety of strains acquired from the environment or arose de novo during infection. We also observed a fairly high frequency of RhlR variants (12.4%), which is similar to the rate of RhlR mutation in other types of infection.^55^ The impact of mutations on RhlR function is less well studied, and it is unclear whether the missense mutations identified in our isolates impair RhlR function. Nonetheless, our findings add to a growing body of evidence that quorum sensing receptor genes are often mutated in *P. aeruginosa* isolates from various environments, including acute infections.

The quorum sensing signal synthase genes were much better conserved than the receptor genes in our isolates. Only one isolate in our collection had a nonsynonymous mutation in *lasI*. Among sequenced *P. aeruginosa* isolates, RhlI is particularly variable at residues 62, 83, and 127. Each of the major alleles at these positions is encoded by at least one laboratory strain where RhlI is known to be functional. Discarding these alleles, there were four isolates in our collection that encoded a RhlI amino acid substitution relative to laboratory strains. Three isolates harbored a P159S substitution, which has previously been shown to be functional.^68^ Thus, the majority of isolates in our collection likely encoded functional quorum sensing signal synthases.

We additionally found variability in genes required for biofilm exopolysaccharide synthesis, with two isolates apparently lacking the genes required for Psl synthesis. Further, we identified within-host variability in *mucA* genotype and mucoid phenotype. Many clinical isolates of *P. aeruginosa*, particularly those isolated from chronic lung infections of people with cystic fibrosis, overproduce alginate resulting in a mucoid phenotype. In these isolates, alginate forms a capsule that protects *P. aeruginosa* from antibiotics and host defenses.^59^ The conversion to a mucoid phenotype is primarily driven by mutations in *mucA*. In our collection, 10% of isolates encoded a *mucA* mutation (10 isolates from eight unique patients). Two patients from whom we obtained multiple isolates harbored a mixture of MucA-variant, mucoid isolates, as well as non-mucoid isolates with WT MucA sequences. The isolates from these patients shared a high ANI, suggesting a clonal infection with mutations potentially acquired during infection.

Finally, we assessed both phenotypic and genotypic antibiotic resistance in our isolates. Fluoroquinolones, as well as the aminoglycoside tobramycin and the beta-lactam ceftazidime, are common antibiotic therapies for bacterial keratitis caused by *P. aeruginosa*.^69^ Likely due to the community origins of our collection, we found low rates of resistance in our ocular *P. aeruginosa* isolates. The low rates of fluoroquinolone resistance in our population were comparable to findings in the Antibiotic Resistance Monitoring in Ocular micRoorganisms (ARMOR) surveillance study conducted in the United States from 2009 to 2019, which revealed that fewer than 10% of *P. aeruginosa* isolates were resistant to fluoroquinolones.^70^ Likewise, aminoglycoside resistance in our isolates was also consistent with that from the ARMOR program, which found <3% resistance to tobramycin.^70^ Although in our population we found only two MDR *P. aeruginosa* isolates, keratitis and endophthalmitis caused by MDR *P. aeruginosa* has been reported.^26^^,^^71^^,^^72^ Outside of the United States, resistance rates for *P. aeruginosa* causing eye infections are higher, such as in India where 40% to 60% of isolates of *P. aeruginosa* were found to be resistant to cephalosporins, 40% to 55% to fluoroquinolones, and 30% to 60% to aminoglycosides.^3^ Patient populations at greatest risk of acquiring infections caused by MDR *P. aeruginosa* include those admitted to the intensive care unit in the preceding year, immunocompromised patients, and those previously exposed to antipseudomonal carbapenems and fluoroquinolones.^73^

We detected 14 antimicrobial resistance-associated genes in our ocular *P. aeruginosa* population capable of conferring resistance through various mechanisms including antibiotic inactivation, *aph(3′)-IIb*, *ant(2″)-Ia*, *aph(3″)-Ib*, *aph(6)-Id*, *ant(3″)*, *fosA*, *blaOXA-50*, *blaPAO*, and *catb7*; antibiotic target replacement, *dfrA* and *sul1*; and drug efflux, *tet(C)* and *tet(M)*. However, only five antimicrobial resistance genes—*aph(3′)-llb*, *blaOXA-50*, *blaPAO*, *catb7*, and *fosA*—were present in almost all of our isolates. These are common resistance genes found in *P. aeruginosa*.^25^^,^^74^ *aph(3′)-llb* is a chromosomally encoded aminoglycoside phosphotransferase that confers resistance to a few aminoglycosides,^75^ including kanamycin and neomycin, but not to amikacin, tobramycin, and gentamicin, thus explaining the low level of resistance that we observed phenotypically for these antibiotics. In addition, the presence of *blaOXA-50* and *blaPAO* resistance genes does not confer high levels of resistance to carbapenems and other beta-lactam antibiotics, as resistance is complex and multigenic.^76^ We acknowledge that organism and susceptibility profiles can be geographically specific. Thus, because all isolates in our study were from the New England area, this limits the generalizability of our findings to other geographical regions with different environmental factors, patient populations, healthcare systems, or antibiotic usage patterns.

Overall, we found diversity in *P. aeruginosa* isolates that cause ocular infection with no clear predominance of sequence type or virulence factor alleles. Understanding the genomic diversity of *P. aeruginosa* is crucial for better understanding the variations in pathogenesis between strains and the underlying mechanisms of antibiotic resistance, ultimately helping to guide the management of these infections.

## Supplementary Material

Supplement 1

Supplement 2

Supplement 3

Supplement 4

Supplement 5

Supplement 6
