# Supplementary material for: Whole Genome Analysis of Ocular Pseudomonas aeruginosa Isolates Reveals Genetic Diversity
Source: Invest Ophthalmol Vis Sci. 2025 Jun 18;66(6):58. doi: 10.1167/iovs.66.6.58 (PMC12180601; doi:10.1167/iovs.66.6.58)
Supplement: Supplement 1 [file iovs-66-6-58_s001.pdf]

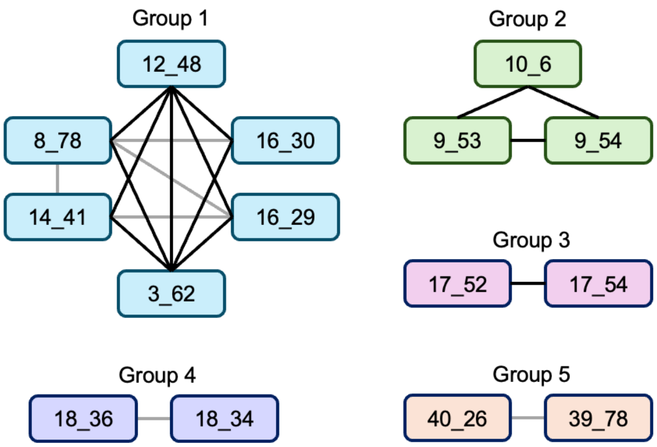

Supplementary Figure 1. Average nucleotide identity (ANI) analysis between pairs of isolates shows isolates collected from a single patient group into clonal clusters. Isolates connected by black lines share >99.99% ANI and isolates connected by grey lines share >99.98% ANI.
